# Evaluación del cambio de luminosidad y sensibilidad en el aclaramiento dental láser asistido: Ensayo clínico controlado aleatorizado

**DOI:** 10.21142/2523-2754-1302-2025-239

**Published:** 2025-05-16

**Authors:** Erica de Jesús, Victoria Rojas, Carlos Sánchez-Ramírez, Javier Vidal, Luisa Araujo, Oriana Parucho, Ricardo Brito, Patricia Moreno-Garcés, Daniela Machado, Roba Izzeddin

**Affiliations:** 1 Facultad de Odontología, Universidad José Antonio Páez. Valencia, Venezuela. ericadejesusdeleca@gmail.com, vmrs.1712@gmail.com, odcarlossanchez@gmail.com, orianaparucho@mail.com, machadogallegosdaniela@gmail.com Universidad José Antonio Páez Facultad de Odontología Universidad José Antonio Páez Valencia Venezuela ericadejesusdeleca@gmail.com vmrs.1712@gmail.com odcarlossanchez@gmail.com orianaparucho@mail.com machadogallegosdaniela@gmail.com; 2 Facultad de Odontología, Universidad Central de Venezuela, Caracas, Venezuela. javiervidalacevedo@gmail.com, araujoaluisam@gmail.com Universidad Central de Venezuela Facultad de Odontología Universidad Central de Venezuela Caracas Venezuela javiervidalacevedo@gmail.com araujoaluisam@gmail.com; 3 Pontificia Universidad Javeriana. info.rbrito@gmail.com Pontificia Universidad Javeriana Pontificia Universidad Javeriana Colombia info.rbrito@gmail.com; 4 Catedra de Radiología, Universidad Central de Venezuela. Caracas, Venezuela. tuortodonciaconpmg@gmail.com Universidad Central de Venezuela Catedra de Radiología Universidad Central de Venezuela Caracas Venezuela tuortodonciaconpmg@gmail.com; 5 Facultad de Odontología, Universidad de Carabobo. Valencia, Venezuela. robaizzeddin@gmail.com Universidad de Carabobo Facultad de Odontología Universidad de Carabobo Valencia Venezuela robaizzeddin@gmail.com

**Keywords:** estética dental, aclaramiento dental, luminosidad del color, sensibilidad dental, pigmentaciones extrínsecas, Dental esthetics, tooth whitening, color luminosity, dental sensitivity, extrinsic pigmentation

## Abstract

**Objetivo:**

Evaluar la efectividad del aclaramiento dental láser asistido sobre la luminosidad del color y la sensibilidad dental.

**Métodos:**

Veintiocho pacientes, un 75% mujeres y un 25% hombres; edad media: 21,75 años fueron divididos aleatoriamente en dos grupos: experimental y control (n = 14). Se utilizó peróxido de hidrógeno al 35% en ambos grupos. En el grupo experimental, el gel se activó con un láser diodo de 980 nm y densidad de energía de 86,41 J/cm² (tres aplicaciones), mientras que el grupo control no recibió láser. Se realizaron fotografías estandarizadas para analizar la luminosidad en el tercio medio del incisivo central superior derecho, usando coordenadas CIELab en tres momentos: pretratamiento, postratamiento inmediato y a los 7 días. La sensibilidad dental se evaluó mediante una escala visual análoga. Los datos se analizaron con pruebas U de Mann Whitney y t de Student. (p < 0,05).

**Resultados:**

No hubo diferencias significativas en la luminosidad postratamiento inmediato entre grupos (p = 0,412), pero sí a los 7 días (p = 0,000), donde el grupo láser mostró mayor estabilidad del color. En cuanto a la sensibilidad, aunque no fue significativa (p > 0,05), los valores a 1 y 24 horas fueron menores en el grupo experimental, indicando menor hipersensibilidad.

**Conclusión:**

El aclaramiento láser asistido con diodo de 980 nm mejora la estabilidad de la luminosidad del color a los 7 días, lo que ofrece beneficios clínicos significativos. Aunque la sensibilidad no mostró diferencias estadísticamente significativas, el menor malestar reportado en el grupo láser destaca su potencial para mejorar la experiencia del paciente.

## Abstract (en)

**Objective:**

To evaluate the effectiveness of laser-assisted dental bleaching on color luminosity and dental sensitivity.

**Methods:**

Twenty-eight patients (75% female, 25% male) with a mean age of 21.75 years were randomly allocated into two groups: experimental and control (n = 14). Both groups received 35% hydrogen peroxide. In the experimental group, the bleaching gel was activated with a 980 nm diode laser at an energy density of 86.41 J/cm² (three applications), whereas the control group did not receive laser activation. Standardized photographs were taken to evaluate luminosity at the middle third of the right maxillary central incisor, using CIELab coordinates at three time points: baseline, immediately after treatment, and at 7 days post-treatment. Dental sensitivity was assessed using a visual analog scale (VAS). Data were analyzed using Mann-Whitney U and Student’s t-tests (p < 0.05). 6

**Results:**

No statistically significant differences in immediate post-treatment luminosity were found between groups (p = 0.412); however, significant differences were observed at 7 days (p = 0.000), with the laser group exhibiting greater color stability. Although sensitivity differences were not statistically significant (p > 0.05), lower VAS scores at 1 and 24 hours were recorded in the experimental group, indicating reduced hypersensitivity.

**Conclusion:**

Laser-assisted bleaching with a 980 nm diode laser improves the stability of color luminosity at 7 days, offering clinically relevant benefits. While sensitivity did not show statistically significant differences, the reduced discomfort reported in the laser group highlights its potential to enhance the overall patient experience.

## INTRODUCCIÓN

**Figura 1:**
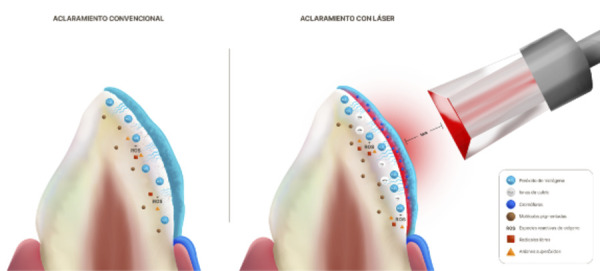
Mecanismo de acción del aclaramiento dental convencional y aclaramiento dental láser asistido. A) Aclaramiento convencional. El H_2_O_2_ penetra el esmalte y llega a la dentina, lo que libera ROS, radicales libres y aniones superóxidos que interactúan con las moléculas pigmentantes. B) Aclaramiento láser asistido. La luz láser presenta afinidad hacia los cromóforos presentes en el gel y activa el H2O2, lo que genera una mayor penetración y liberación de ROS, radicales libres y aniones superóxidos que interactúan con las moléculas pigmentantes. Esto afecta en menor proporción la microdureza del esmalte y se observa una mayor cantidad de iones de calcio en comparación con el aclaramiento convencional (^30^).

El aclaramiento dental tiene un gran impacto en la apariencia de la estructura dental, al atenuar las pigmentaciones intrínsecas que ocasionan insatisfacción en los pacientes ^1^. Por ello, dicho tratamiento es uno de los más aceptados y requeridos, ya que se considera un procedimiento eficaz, seguro y no invasivo ^2^. El agente activo del aclaramiento dental conocido como peróxido de hidrógeno (H2O2) es una sustancia inestable de bajo peso molecular que penetra en la estructura dental, se descompone y libera especies reactivas de oxígeno (ROS), radicales libres y aniones superóxidos que tienen la capacidad de difundirse a través del esmalte y la dentina, lo que provoca la oxidación de las moléculas pigmentadas que provocan el oscurecimiento de la estructura dental ^3^. Por su parte, el H2O2, a través de las ROS liberadas, actúa sobre los electrones de largas cadenas moleculares de los cromóforos y produce la oxidación de las macromoléculas pigmentadas en moléculas menores e hidrosolubles. Esto tiene un efecto aclarador sobre el color dental, lo que altera su percepción visual ^4^ (Figura 1A).

Para definir el color, la Commission Internationale de lÉclairage (CIE) creó un espacio de color denominado CIE LAB, al correlacionar los valores numéricos de color consistentemente con la percepción visual humana, cuyas coordenadas se indican con las siglas L*, a* y b*. L* representa la luminosidad del color y tiene un límite numérico que va desde el negro (0) hasta el blanco (100), mientras que a* y b* representan la cromaticidad (matiz y saturación) y no tienen un límite numérico específico. Asimismo, el valor a* negativo corresponde al verde, mientras que el a* positivo corresponde al rojo; y un valor b* negativo corresponde al azul, mientras que el b* positivo corresponde al amarillo ^5^.

Según diversos autores ^5^^,^^6^, L* es el parámetro más importante, ya que el ojo humano lo observa y percibe mayormente con respecto a los otros parámetros del color. Su toma se fundamenta en el uso de la fotografía digital, los espectrómetros, espectrorradiómetros y colorímetros para abordar la medición y comparación del color; por ello, el método universal corresponde al uso del espacio de color CIE LAB, a través de las coordenadas obtenidas ^7^.

Asimismo, en la búsqueda constante de optimizar y mejorar los tratamientos odontológicos se han planteado diversos métodos para potenciar el aclaramiento dental, entre ellos, los láseres diodo de diversas longitudes de onda ^8^. Su uso está sujeto a que estos presentan afinidad con los cromóforos presentes en el gel aclarador, por lo cual los activa y genera una disociación más rápida del H2O2, lo que aumenta la liberación y penetración ROS, radicales libres y aniones superóxidos que interactúan con las moléculas pigmentantes presentes en la dentina, sin aumentar la temperatura de forma tal que pueda afectar la pulpa dental ^9^ (Figura 1B).

Un estudio ^10^ reportó que el aclaramiento activado por láser es más eficaz que el convencional. Por otro lado, en una investigación similar ^11^ evaluaron la eficacia del aclaramiento dental convencional en comparación con el láser asistido, para lo cual utilizaron tres longitudes de onda (810, 940 y 980 nm), las cuales no mostraron diferencias en términos de eficacia, con resultados similares en cuanto al cambio de color respecto del grupo control; sin embargo, la activación láser disminuyó el tiempo de trabajo del tratamiento. Por otro lado, diversos autores ^12^ reportaron que la fotoactivación con láser de diodo del gel aclarador resultó ser más eficiente, y que la longitud de onda de 940 nm fue la más eficaz para lograr el cambio de color y presentó un aumento mínimo en la temperatura intrapulpar. Asimismo, en una revisión sistemática ^13^ se concluyó que el láser diodo con una longitud de onda entre 790 y 980 nm es capaz de reducir el sobrecalentamiento del órgano dental, lo que implica niveles de sensibilidad dental disminuidos. Por el contrario, se ha reportado que la activación con láser del agente aclarador promueve la eficacia del tratamiento, pero no reduce la sensibilidad posoperatoria ^14^. Es importante considerar que las variaciones en los parámetros del dispositivo láser, la técnica de aplicación (enfocada o desenfocada), el diámetro del *spot* y ciertas características del tejido dental pueden influir en la interacción láser-tejido y, por ende, en los resultados del tratamiento.

El objetivo de la presente investigación fue evaluar el cambio de la luminosidad y la sensibilidad dental en el aclaramiento dental con láser asistido. La hipótesis nula se establece como sigue: i) el láser no aumenta significativamente la estabilidad del color y ii) no reduce la sensibilidad postratamiento.

## MATERIALES Y MÉTODOS

### Diseño del estudio

**Figura 2:**
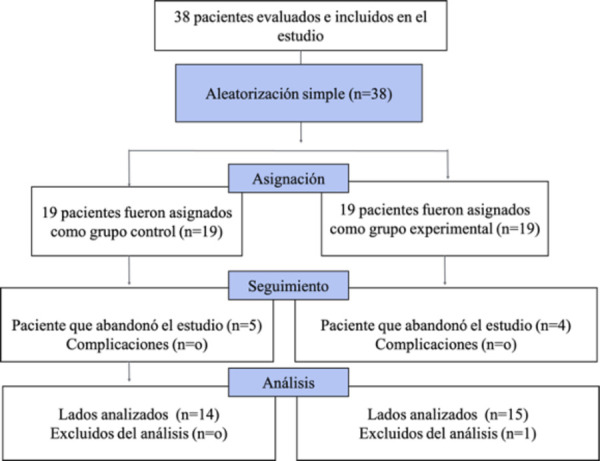
Diagrama de flujo CONSORT. Se incluyeron 38 pacientes en el estudio; sin embargo, 5 pacientes del grupo control abandonaron el estudio tras el procedimiento de aclaramiento dental, y 5 pacientes del grupo experimental no asistieron a los controles postratamiento. Debido a esto, fueron excluidos del análisis, lo que dio como resultado una muestra final de 28 pacientes.

Para abordar el propósito de la investigación, los investigadores diseñaron e implementaron un ensayo clínico controlado aleatorizado. El trabajo se realizó en la clínica integral de la Facultad de Odontología de la Universidad José Antonio Páez, entre abril y junio de 2024, y fue aprobado por el Comité de Bioética de la Facultad de Odontología de la Universidad Central de Venezuela, con el número CB-219-2024. Asimismo, se registró en Open Science Framework OSF (doi 10.17605/OSF.IO/DUPA3). La asignación de los grupos experimental y control fue realizada mediante una técnica de aleatorización simple, usando el *software* libre www.sealedenvelope.com (cuyos resultados fueron introducidos en sobres opacos y sellados por un investigador no implicado en la ejecución del tratamiento). Siguiendo los estándares consolidados de informes de ensayos (www.consort-statement.org) (Figura 2) y los principios de la Declaración de Helsinki ^15^. 

### Cálculo del tamaño de la muestra

Estudios anteriores demostraron que el uso de un agente aclarador con un 35% de H2O2, activado o no con láser diodo conduce a un valor de L* de 7,0 a 2,0 después de dos o tres sesiones de aclaramiento ^11^^,^^16^^,^^17^. Para tener un 90% de probabilidad de significancia al nivel del 5%, y considerando un cambio en la medida de resultado primaria de 7 en el grupo de control a 5 en el grupo experimental, se estimó una muestra mínima de 28 pacientes.

### Criterios de inclusión

Pacientes de ambos sexos, saneados, que presentaron indicación para aclaramiento dental, en edades comprendidas entre 18 y 30 años.

### Criterios de exclusión 

Pacientes con restauraciones anteriores o prótesis dentales, con aparatología de ortodoncia, pigmentaciones por tetraciclinas, fluorosis y asociadas a dientes no vitales. Asimismo, pacientes con hipersensibilidad dental o cualquier otra patología que pueda causar sensibilidad (migración gingival, exposición dentinaria y fisura dental), pacientes bajo medicación con antiinflamatorios o analgésicos, fumadores, bruxómanos, con enfermedad periodontal y/o caries dental, pacientes con antecedentes de procedimientos de aclaramiento dental. Además, fueron excluidas mujeres en la fase lútea de su ciclo menstrual (10 días que anteceden su próxima menstruación), debido a que este altera la percepción de la sensibilidad dental, así como embarazadas o lactantes. Estos datos fueron tomados tras una anamnesis y examen clínico realizado por uno de los investigadores.

### Protocolo del tratamiento

#### Grupo control

Se realizó el aclaramiento dental con H2O2 al 35% (Pola Office, SDI), modificando el protocolo de la casa comercial ^18^. Para ello, se aplicó una capa de 2 mm de grosor en las caras vestibulares desde el segundo premolar derecho hasta el segundo premolar izquierdo, en ambas arcadas. El gel se dejó actuar durante de 8 minutos y se realizaron 3 aplicaciones con un intervalo de 2 min entre ellas. Luego del tiempo preestablecido, se retiró el gel aclarador con una cánula de succión y se lavó con abundante agua por 2 minutos. 

#### Grupo experimental

**Figura 3:**
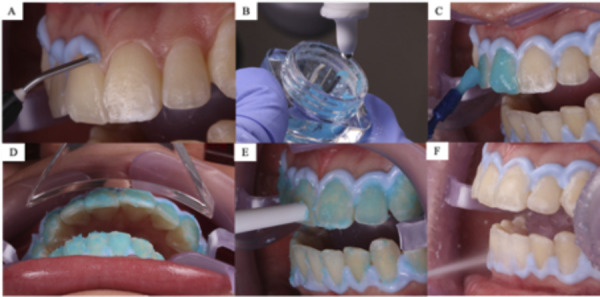
Protocolo de tratamiento en grupo experimental. A) Aplicación de barrera gingival. B) Preparación del gel aclarador. C) Aplicación de agente aclarador. D) Irradiación con láser diodo de 980 nm. E) Succión del gel aclarador. F) Lavado con agua del gel aclarador.

**Figura 4:**
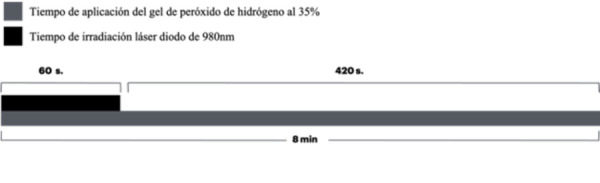
Ilustración esquemática del ciclo de protocolo de irradiación láser

Se utilizó el mismo protocolo del grupo control, con la diferencia de que se empleó un láser diodo 980 nm (PIOON Unilase) programado según el protocolo de Al-Karadaghi *et al*. ^12^, a una potencia de 7 watts en modo continuo. Se utilizó una pieza de mano con un área de 4,86 cm^2^, se colocó a una distancia de 1cm y se dejó actuar durante 60 s (Figura 3). Este procedimiento se ejecutó al primer minuto de cada sesión, con un periodo de descanso entre sesiones de 2 min, lo que logró controlar el aumento de la temperatura dental (Figura 4). La dosificación de la densidad de energía se calculó según la fórmula dE= E/A ^10^ (Tabla 1). Como resultado, se obtuvieron las siguientes densidades de energía utilizadas: 

dE = E/A dE = Densidad de energía E = energía (420) A = área (4,86 cm^2^)

dE = 86,41 J/cm^2^

**Tabla 1:** Parámetros de irradiación del láser

Potencia (W/CW)	Área (cm^2^)	Energía (J)	Tiempo (s)	Densidad de energía (J/cm^2^)	Total, de sesiones	Total, de irradiación emitida (J/cm^2^)
7	4,86	420	60	86,41	3 X (60 seg)	259,23

W: continuo, CW: pulsado, cm^2^: centímetros cuadrados, J: Joules, s: segundos, J/cm^2^: Joules sobre centímetros cuadrados.

### Evaluación de luminosidad (L*): análisis fotográfico

Las fotografías fueron tomadas con una cámara digital Canon EOS Digital Rebel T2i, con un macro de 100 mm (EF 100 mm f/2.8 LC Macro IS USM, Canon), ring flash MR-14EX con un filtro de polarización cruzada, en el modo manual, con las siguientes características: velocidad de obturación de 1/125 seg, apertura de f22, ISO 100, en formato RAW. El flash externo anular o lateral se ajustó a la máxima potencia (1/1).

El protocolo fotográfico consistió en retraer labios y carrillos con retractores plásticos. Luego, se posicionó la tarjeta de grises (White Balance, Emulation) con la línea negra vertical que la divide coincidiendo con la línea media dentaria; el semicírculo más grande de la tarjeta coincidió con el de la cámara, ya que el tipo de sensor es de fotograma completo. Las fotografías se realizaron con luz natural en el horario comprendido entre las 10:00 a. m. y 12:00 m., según el protocolo Digital Smile Design (DSD) ^19^, a 50 cm de distancia de los dientes.

La medición de L* se ejecutó con el método fotocolorimétrico digital (PCM), el cual consiste en el uso de una cámara réflex digital de lente única (DSRL), un flash macro, un objetivo macro y un filtro de polarización cruzada, para la posterior formulación del color ^20^.

Tras la obtención de las fotografías, se importó el archivo RAW en Adobe PhotoShop (2021 22.5.1 Windows 11), se realizó el balance de blancos con la tarjeta de grises y la corrección de brillo o balance de exposición, con las funciones integradas del programa. Esto se llevó a cabo hasta que el valor de L* conocido (L*79) de la tarjeta de grises fue reproducido, es decir, 0,12. Seguidamente, para obtener las coordenadas de color del diente en el espacio de color CIE L*a*b* que se ha usado ampliamente en la literatura ^10^^,^^17^, se utilizó el *software* Classic Color Meter (versión 1.8.1 para MacIntosh AC; Ricci Adams) ^20^. En la presente investigación, se tomó en cuenta únicamente el parámetro L*.

El tamaño de la apertura de medición se ajustó para cuantificar el color del diente en el área de interés, la cual fue el tercio medio del incisivo central superior derecho. Se midió el color en 3 ocasiones: pretratamiento, postratamiento (inmediato) y a los 7 días postratamiento, para evaluar la estabilidad de L*. El evaluador de L*, no tuvo acceso a la información sobre la asignación de los pacientes, lo que permitió minimizar el sesgo de detección.

### Sensibilidad dental

Para medir la sensibilidad dental postratamiento, se utilizó EVA ^21^, en cuyos extremos se encontraron las expresiones del dolor, donde 0 o extremo izquierdo representó el estado sin dolor y 10, el extremo derecho, representó la mayor intensidad del dolor. Dicha sensibilidad se tomó en cuenta tras los primeros 60 min, 24 min, 48 min y 72 horas postratamiento.

### Análisis estadístico 

Los datos de las variables fueron analizados mediante el paquete estadístico SPSS 15.0 (SPSS IMB Statistics. IMB Corporation, EE. UU). Las variables categóricas (género) fueron descritas mediante tablas de frecuencias y porcentajes. Las variables numéricas se describieron mediante estadísticos de tendencia central: valor medio, desviación estándar, máximo y mínimo. Las estadísticas de contraste se realizaron usando las pruebas de T de student y U de Mann Whitney, de acuerdo con la normalidad de los datos. 

## RESULTADOS

En la Tabla 2, se muestran las características de los pacientes evaluados. La edad media fue de 21,75 con una mínima de 18 y una máxima de 30 años, así como una desviación estándar de 2,5. De los 28 pacientes, el 75% (21) eran femeninos y el 25% (7), masculinos.

**Tabla 2:** Características iniciales de los pacientes evaluados

Características	
Pacientes evaluados: n	28
Edad: media ± DE	21,75 ± 2,51
Rango	18-30
Género:	f (%)
Masculino	7 (25,0)
Femenino	21 (75,0)

f: frecuencia, DE: desviación estándar

**Figura 5:**
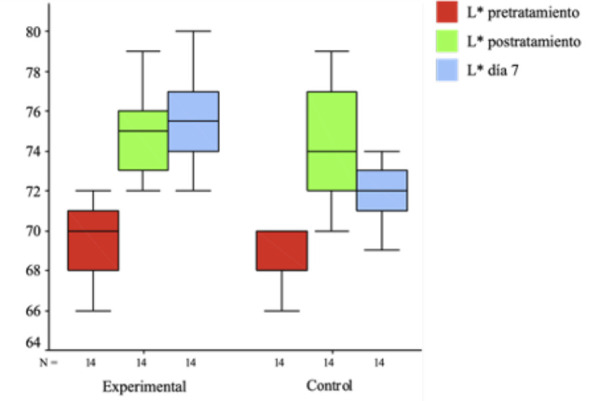
Valores medios de la L* previa, postratamiento inmediato y a los 7 días para grupos láser y control. Las diferencias intragrupos se evaluaron mediante la prueba T de Student con diferencias medias para muestras independientes.

En la Figura 5, se muestran comparativamente los valores medios de L* pre, post inmediato y a los 7 días, para los grupos experimental y control. Las medias en L* del grupo control variaron entre 69,0, 74,3 y 72,2 en las evaluaciones previas, post inmediato y a los 7 días, respectivamente. El incremento de L* post inmediato en el grupo control fue del 7,6 %, mientras que al día 7 fue del 4,6%. 

Las medias de L* en el grupo experimental variaron entre 69,6, 75,1 y 75,8 en las evaluaciones previa, post inmediato y a los 7 días, respectivamente. El incremento en L* post inmediato en el grupo láser fue del 8% y al día 7 fue del 9%.

La diferencia entre las medias de L* entre el grupo experimental y control no fue significativa en la medición de L* pretratamiento (*p* = 0,46). Asimismo, la diferencia entre las medias de L* postratamiento inmediato de los grupos experimental y control no fue estadísticamente significativa (*p* = 0,412). Sin embargo, la diferencia entre las medias de L* a los 7 días fue de los grupos experimental y control, estadísticamente significativas (*p* = 0.000).

**Tabla 3:** Prueba de contraste T student de diferencias de medias para muestras independientes entre L* pre, post inmediata y a los 7 días para grupo experimental y control

	Grupo	N	Media	DE	p
L* pretratamiento	Experimental	14	60,6	1,9	0,469
	Control	14	60,0	2,6	
L* post inmediato	Experimental	14	75,1	2,0	0,412
	Control	14	74,2	2,8	
L* a los 7 días	Experimental	14	75,8	2,5	<0,001
	Control	14	72,2	2,2	

L*: luminosidad, N: número, DE: desviación estándar

Prueba T de Student (p < 0,05)

En cuanto a la sensibilidad dental, las diferencias de las medias no fueron significativas para ninguna de las dos comparaciones (Tabla 4); sin embargo, el grupo experimental presentó valores más bajos de sensibilidad dental que el grupo control. Es importante mencionar que los pacientes de ambos grupos, para las evaluaciones de las 48 y 72 horas, no presentaron sensibilidad alguna. Aunque no hubo diferencias significativas, el grupo láser reportó menor incomodidad, lo que sugiere un beneficio clínico importante para el confort del paciente.

**Tabla 4:** valores de la media de la sensibilidad en los grupos experimental y control por sesión de tratamiento

	Grupo	N	Media	DE	p
Sensibilidad 1h post	Experimetal	14	3,50	2,4	0.495
	Control	14	3,79	2,12	
Sensibilidad 24 h post	Experimental	14	0,93	1,64	0,350
	Control	14	1,14	1,41	
Sensibilidad 48 h post	Experimental	14	-	-	-
	Control	14	-	-	-

N: número, DE: desviación estándar

Prueba U de Mann Whitney, (p < 0,05)

## DISCUSIÓN

En la literatura existen diversas fuentes de luz para la activación del gel en el aclaramiento dental; sin embargo, son evidentes las controversias en sus protocolos. La fuente de luz propuesta en la actualidad para la activación del gel aclarador es el láser de diodo ^22^.

El protocolo empleado en la presente investigación fue similar al utilizado por otros autores ^12^, quienes trabajaron con una longitud de onda de 980 nm, a una potencia de 7 watt, en modo continuo. La irradiación se ejecutó durante 120 s, en dos sesiones. Se reportó que el aumento de la temperatura intrapulpar fue seguro, lo cual respalda los resultados encontrados en este estudio. A pesar de que no se evaluó la temperatura intrapulpar, no se apreció ninguna complicación ni alteración de la misma, lo que concuerda con otro estudio similar ^23^ que obtuvo resultados semejantes.

Asimismo, diversos autores ^8^^,^^24^ sugieren que, en dicho tratamiento, al emplear el láser de diodo, es importante considerar características como el suministro continuo versus el pulsado, la frecuencia del pulso y su duración; así como otras variables que se relacionan con el método de transferencia de energía, entre ellas el modo de entrega con contacto o sin contacto, enfocado o no, y el diámetro del *spot*. Por consiguiente, recomiendan que la pieza de mano debía usarse sin contacto sobre el área irradiada, desenfocada y con emisión continua, debido a que la absorción en el gel aclarador es mayor; así, la activación se produce de manera efectiva. Este procedimiento concuerda con el de la presente investigación. 

Es crucial destacar que la dosis de energía es fundamental para garantizar la eficacia del tratamiento; por lo tanto, es necesario calcular meticulosamente la densidad de energía en relación con el área irradiada. Este aspecto representa uno de los principales desafíos al comparar los estudios revisados, ya que no todos logran realizar este cálculo de manera precisa, lo que puede generar parámetros incorrectos y resultados impredecibles. Deben considerarse las diferencias en el tiempo de exposición del gel a la luz láser y el protocolo de aclaramiento específico empleado, así como el color y pigmentos presentes en el gel aclarador ^25^. 

En el presente estudio, se utilizó una densidad de energía de 86,41 J/cm^2^ por sesión, con un total de 259,23 J/cm^2^ en el tratamiento, difiriendo con un estudio ^26^ en donde utilizaron una densidad de energía de 30 J/cm^2^ y un láser de 808 nm, y con otros autores ^10^, quienes emplearon diversas longitudes de onda y densidades de energía que oscilaron entre 53, 106,10, 105 y 47,16 J/cm^2^. En dichos estudios, los resultados más eficaces en cuanto al cambio del color dental correspondieron al láser de 940 nm y fluencia de 105 J/cm^2^, y al láser de 445 nm con fluencia de 53 J/cm^2^. Esto podría explicar el menor aumento de L* en nuestro estudio. Al respecto, la literatura consultada ^10^^,^^26^ reporta que se deben usar densidades de energía entre 30 y 106,10 J/cm^2^; por lo tanto, se recomienda oscilar entre esas densidades en investigaciones futuras, para así determinar si existe un mayor aumento de L*.

La pigmentación del gel influye en la cantidad de energía absorbida, la acción del láser y su efectividad en el aclaramiento; por tal motivo, en esta investigación se utilizó un gel de color azul (Pola Office, SDI), debido a que el láser de 980 nm tiene afinidad por los cromóforos azules/violetas ^25^ y una mayor absorción de la luz láser en el gel aclarador. Otros investigadores ^12^ tomaron en consideración este aspecto y potenciaron la acción del gel aclarador al mezclarlo con 0,2 ml de pigmento violeta ultramar, y el uso de un láser de 980 y 940 nm. Por tanto, la presencia de pigmentos afines al tipo de luz láser proporciona una mejor absorción de esta luz en el gel, con una menor transmisión de energía hacia el tejido pulpar ^8^. Un parámetro tomado en cuenta en este estudio fue que el grosor de la capa del gel de aclaramiento debía ser de 2 mm, aproximadamente, para que así la energía se concentre y la reacción con el cromóforo se potencie. 

Para la evaluación del color dental es importante mencionar que su percepción es una experiencia subjetiva y la evaluación por parte del ser humano está influenciada por variables ambientales, biológicas y físicas. Por esa razón, en el presente estudio se midieron los parámetros de color CIE L*a*b*, y se tomó en cuenta únicamente los valores del eje L*. Diversos autores ^5^^,^^6^ mencionan que la L* es el parámetro más observado por el ojo humano con respecto a los otros parámetros del color, lo cual se debe a que la calidad de las células de bastón fotorreceptoras responsables de la detección de los colores blanco y negro es mucho mayor que la de las células fotorreceptoras responsables de la visión del color.

Los hallazgos de este estudio señalaron que hubo un aumento en la L* del color en el grupo láser, con respecto al grupo control; no obstante, estos resultados no fueron estadísticamente significativos (p > 0,05) en la evaluación pre y post inmediata; por lo tanto, no se rechazó la primera hipótesis nula. Efectos similares han sido reportados por un estudio ^11^ en el que estableció que el aclaramiento láser asistido con longitudes de onda de 810, 940 y 980 nm tiene una eficacia similar a la del aclaramiento convencional. Aun así, demostraron que el láser disminuyó el tiempo de trabajo. 

Entre las limitaciones de dicho estudio, los autores evaluaron el color dental pre y post inmediato al tratamiento, sin evaluar su estabilidad en el tiempo. 

Autores mencionan ^17^ que la deshidratación del diente durante el proceso del aclaramiento contribuye al cambio de L* inmediato al tratamiento, y recomiendan que el color sea evaluado después de la absorción de agua en el diente. Por tal motivo, en la presente investigación se evalúo la luminosidad dental al día 7 postratamiento, en donde el grupo experimental mostró un aumento de L* y lo mantuvo estable (p = 0,000), por lo que fue estadísticamente significativo, en comparación con el grupo control donde hubo menor estabilidad de L* en el tiempo. En consecuencia, se rechazó la segunda hipótesis nula.

En otro estudio ^27^ se señala que la efectividad del aclaramiento dental y su estabilidad a largo plazo no dependen de la técnica empleada, y que cualquier variación regresiva de color debe ser atribuida a factores extrínsecos como alimentos, hábitos del paciente o la permeabilidad o defectos en el esmalte. Respecto de este último punto, diversos autores ^28^^,^^29^ reportaron alteraciones en la morfología del esmalte, pérdida de contenido mineral, disminución de la microdureza y pérdida de la capa de esmalte aprismático tras la aplicación de H2O2 sin el uso de láser. Estos efectos, evidentemente, no son deseados.

Otras investigaciones ^30^^-^^33^ han reportado cambios importantes en la microdureza del esmalte al comparar la técnica de aclaramiento convencional con la fotoactiva. Específicamente, reportaron que la activación con láser causó alteraciones mínimas o no significativas en el esmalte. Asimismo, el agente aclarador activado por láser no es invasivo, y se observa un aumento significativo en los niveles de calcio posteriormente al aclaramiento láser asistido, y el láser de diodo de 980 nm fue el que brindó mejores resultados en términos de microdureza. Es importante mencionar que los efectos en el esmalte, como la disminución de la microdureza y la pérdida de contenido mineral, que lo hacen más poroso y permeable, podrían estar relacionados con una tendencia de mayor adhesión de sustancias pigmentantes y su difusión del esmalte hacia la dentina, lo que afectaría directamente la estabilidad de L* del color dental postratamiento. Sin embargo, no existen investigaciones que relacionen la microdureza del esmalte con la estabilidad de L* en el tiempo, tras el aclaramiento láser asistido, por lo que se hace difícil encontrar datos certeros, establecer criterios con fundamento biológico y químico para comparar resultados, y afirmar tal relación. De ahí que resulte de gran interés para los autores y deba ser estudiado en futuras investigaciones. 

Con respecto a los resultados de la sensibilidad dental, no fueron estadísticamente significativos (p > 0,05), por lo que se acepta la hipótesis nula III. Sin embargo, en el grupo experimental se observó una disminución de la sensibilidad dental en comparación con el grupo control. Esto podría atribuirse a que la energía del láser se concentra en los cromóforos del gel aclarador, lo que aumenta su potencial de acción y evita la transmisión de la energía en exceso hacia el tejido pulpar. Además, es posible que la radiación de fondo o residual sea entregada a las células presentes a nivel de la pulpa dental a través de un proceso conocido como fotobiomodulación, el cual desencadena una serie de reacciones bioquímicas. Este fenómeno coincide con estudios previos ^17^ que reportaron una disminución de la sensibilidad en el aclaramiento dental láser asistido en comparación con el convencional. Sin embargo, se requieren investigaciones adicionales para confirmar este hallazgo y elucidar los mecanismos involucrados.

El láser diodo de 980 nm demuestra resultados beneficiosos en el aclaramiento dental, si bien estos no son estadísticamente significativos. No obstante, los resultados obtenidos al día 7 demostraron una estabilidad de L* en grupo láser en comparación con el grupo control; no existe un consenso sobre la verdadera eficacia del láser, por ello, este tema debe estudiarse a fondo para evaluar su verdadero valor y evaluación costo-beneficio para el odontólogo y el paciente. Las limitaciones del estudio incluyen la ausencia de evaluación del efecto del láser en relación con la microdureza del esmalte, el grado de satisfacción del paciente, el uso de otras longitudes de onda del láser de diodo y la evaluación a largo plazo (mayor a 7 días) de la estabilidad de la luminosidad del color dental.

El uso del láser en odontología, aunque implica un mayor costo inicial debido a la adquisición del equipo, reduce significativamente los tiempos de tratamiento y mejora la experiencia del paciente. Este costo-beneficio favorece tanto a los profesionales como a los pacientes. La relevancia clínica del láser radica en su capacidad para proporcionar mayor confort al paciente y resultados más predecibles a largo plazo, lo cual podría justificar su adopción más amplia en la práctica odontológica.

## CONCLUSIONES

El aclaramiento dental asistido con láser mostró una mayor estabilidad en la luminosidad (L*) a los siete días postratamiento, con diferencias estadísticamente significativas (p < 0.05), lo que sugiere un beneficio clínico en el trtamiento. Aunque la reducción de la sensibilidad dental no fue significativa, los pacientes tratados con láser reportaron menor incomodidad. Estos hallazgos posicionan al láser como una herramienta prometedora en odontología estética. Sin embargo, se requieren más estudios con mayor tamaño muestral y control de variables clave para optimizar su eficacia y evaluar su impacto a largo plazo.

## Financiamiento:

Autofinanciado
